# Supplementary material for: Metagenomic binning with assembly graph embeddings
Source: Bioinformatics. 2022 Aug 16;38(19):4481–7. doi: 10.1093/bioinformatics/btac557 (PMC9525014; doi:10.1093/bioinformatics/btac557)
Supplement: btac557_supplementary_data [file btac557_supplementary_data.zip › amber outputs.zip!/amber outputs/strong100/genome/graphmb/heatmap_SAMPLEID.pdf]

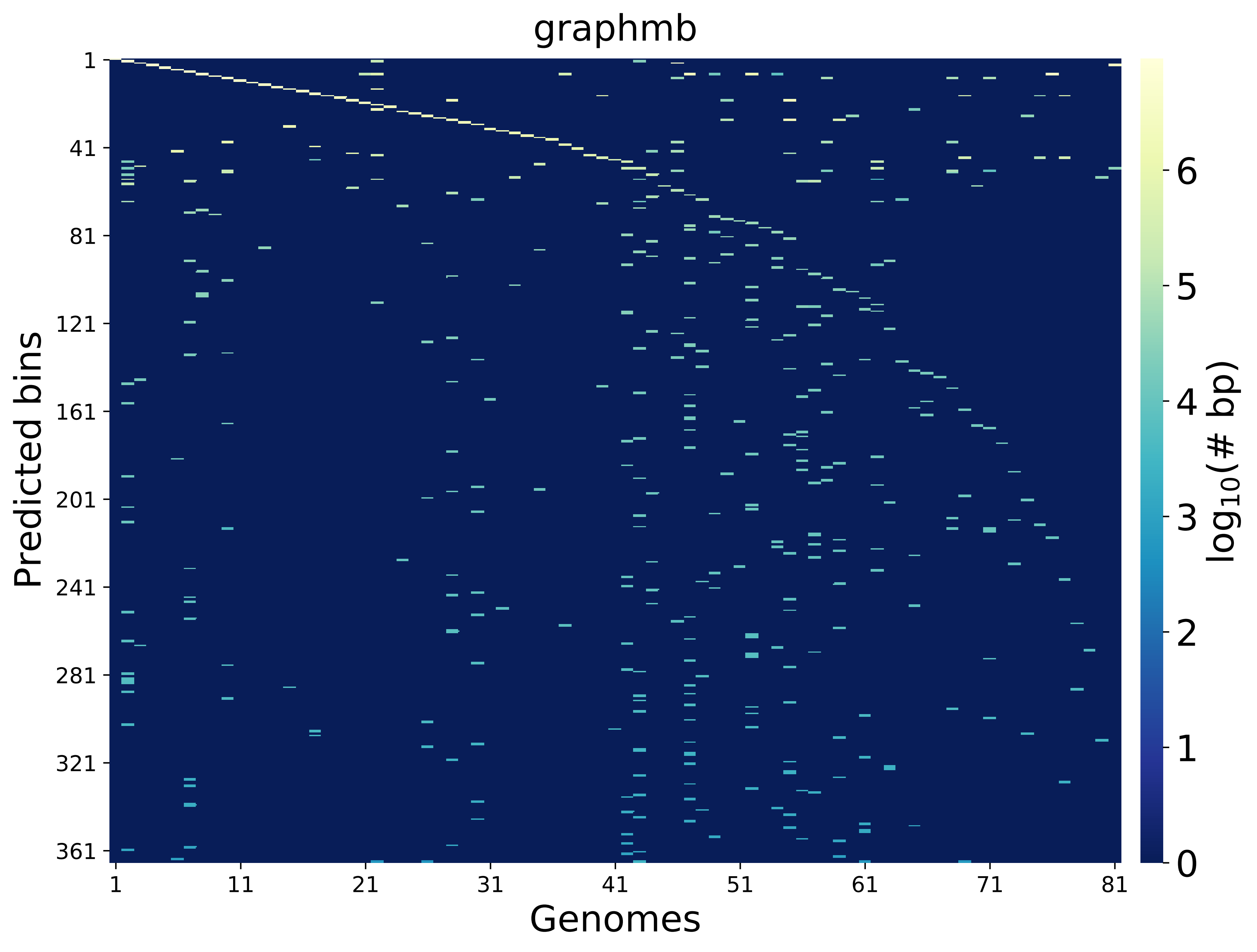
graphmb
Predicted bins
1
41
81
121
161
201
241
281
321
361
Genomes
1
11
21
31
41
51
61
71
81
$\log_{10}$(# bp)
0
1
2
3
4
5
6
